# Porphyrias in the Age of Targeted Therapies

**DOI:** 10.3390/diagnostics11101795

**Published:** 2021-09-29

**Authors:** Angelika L. Erwin, Manisha Balwani

**Affiliations:** 1 Center for Personalized Genetic Healthcare, Cleveland Clinic & Cleveland Clinic Lerner College of Medicine of Case Western Reserve University, Cleveland, OH 44195, USA; 2 Department of Genetics and Genomics, Icahn School of Medicine at Mount Sinai, New York, NY 10029, USA; manisha.balwani@mssm.edu

**Keywords:** porphyria, heme biosynthesis, acute porphyria, cutaneous porphyria, siRNA, small molecule, chaperone, hematin, givosiran, afamelanotide, MT-7117, ciclopirox

## Abstract

The porphyrias are a group of eight rare genetic disorders, each caused by the deficiency of one of the enzymes in the heme biosynthetic pathway, resulting in the excess accumulation of heme precursors and porphyrins. Depending on the tissue site as well as the chemical characteristics of the accumulating substances, the clinical features of different porphyrias vary substantially. Heme precursors are neurotoxic, and their accumulation results in acute hepatic porphyria, while porphyrins are photoactive, and excess amounts cause cutaneous porphyrias, which present with photosensitivity. These disorders are clinically heterogeneous but can result in severe clinical manifestations, long-term complications and a significantly diminished quality of life. Medical management consists mostly of the avoidance of triggering factors and symptomatic treatment. With an improved understanding of the underlying pathophysiology and disease mechanisms, new treatment approaches have become available, which address the underlying defects at a molecular or cellular level, and promise significant improvement, symptom prevention and more effective treatment of acute and chronic disease manifestations.

## 1. Introduction

**Figure 1:**
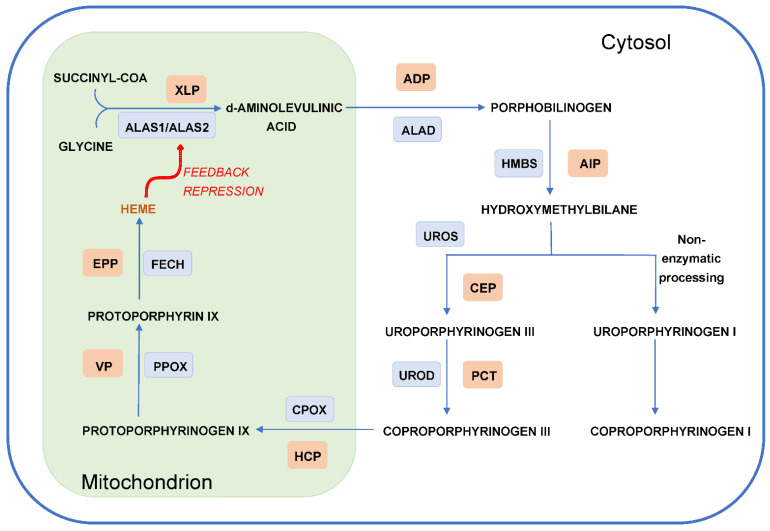
The heme biosynthetic pathway: Eight enzymatic steps lead to the conversion of succinyl-CoA and glycine to the end product, heme, which is then transported out of the mitochondrion and used for the formation of various hemoproteins. Especially in the liver, heme exerts a negative feedback on the first enzyme of the heme biosynthetic pathway, ALAS1. While ALAS1 is ubiquitously expressed, its isoform, ALAS2, is erythroid specific and is regulated by erythroid-specific transcription factors. Four catalytic reactions of the heme biosynthetic pathway occur in the mitochondrion and the other four steps in the cytosol. Dysfunction of each enzyme (in blue boxes) results in a different type of porphyria (in red boxes) due to accumulation of the different heme precursors and porphyrins in various tissues. Abbreviations: enzymes of the heme biosynthetic pathway: ALAS1/ALAS2, delta-aminolevulinic acid synthase 1/2; ALAD, delta-aminolevulinic acid dehydratase; HMBS, hydroxymethylbilane synthase; UROS, uroporphyrinogen III synthase; UROD, uroporphyrinogen decarboxylase; CPOX, coproporphyrinogen oxidase; PPOX, protoporphyrinogen oxidase; FECH, ferrochelatase. Different types of porphyria: XLP, X-linked protoporphyria; ADP, delta-aminolevulinic acid dehydratase deficiency porphyria; AIP, acute intermittent porphyria; CEP, Congenital erythropoietic porphyria; PCT, porphyria cutanea tarda; HCP, hereditary coproporphyria; VP variegate porphyria; EPP, erythropoietic protoporphyria.

The porphyrias are a group of rare inherited metabolic disorders that are caused by deficiencies of specific enzymes involved in the heme biosynthetic pathway (Figure 1) [1]. The symptoms observed in the different types of porphyria result from the excessive accumulation of porphyrins or heme precursors in different tissues (Table 1) [2]. While heme synthesis is present in all cell types, the majority (~80%) occurs in erythroblasts in the bone marrow, followed by ~15% in hepatocytes. Based on the primary source of heme precursor or porphyrin overproduction and accumulation, the porphyrias are classified as either hepatic or erythropoietic [3]. From a clinical perspective, the hepatic porphyrias commonly present with acute neurovisceral symptoms and include acute intermittent porphyria (AIP), hereditary coproporphyria (HCP), variegate porphyria (VP) and delta-aminolevulinic acid dehydratase deficiency porphyria (ADP). Erythropoietic porphyrias, which include erythropoietic protoporphyria (EPP), X-linked protoporphyria (XLP), and congenital erythropoietic porphyria (CEP), are characterized mainly by cutaneous manifestations due to phototoxicity. This difference in clinical presentation explains an additional grouping into either acute or cutaneous porphyrias, with two types (HCP and VP) falling into both categories (Table 1). In addition, two types of hepatic porphyrias, porphyria cutanea tarda (PCT) and hepatoerythropoietic porphyria (HEP), present mainly with cutaneous symptoms and do not have neurovisceral involvement [4].

With the exception of PCT, which in most cases is sporadic, porphyrias are caused by pathogenic variants in the genes encoding the different enzymes involved in the heme biosynthetic pathway. Inheritance can be autosomal dominant, autosomal recessive, or X-linked. Especially in the acute hepatic porphyrias, disease penetrance and severity are significantly influenced by a combination of genetic, environmental and physiologic factors, which can lead to disturbance of the tightly regulated heme biosynthesis [5].

### Heme Biosynthesis

Heme is the end product of a metabolic pathway that involves eight enzymatic steps (Figure 1). The first and rate-limiting step is the formation of delta aminolevulinic acid (ALA) from glycine and succinyl-Coenzyme A [1,4]. This step is catalyzed by the enzyme ALA-synthase (ALAS), which has two isoforms—the ubiquitously expressed ALAS1 and the erythrocyte-specific ALAS2. These two different isoforms are encoded by two separate genes—*ALAS1*, which is located on chromosome 3, and *ALAS2*, on the X-chromosome [6,7]. The first and the last three enzymatic steps of the pathway are located in the mitochondrion, whereas the other four enzymes occur in the cytoplasm (Figure 1). The first two catalytic reactions in the pathway lead to the formation of heme precursors delta aminolevulinic acid (ALA) and porphobilinogen (PBG), followed by the production of porphyrin metabolites. These intermediate substrates do not have a physiologic function and do not accumulate under normal conditions. The final step in the pathway leads to the synthesis of heme, which is then further used for the formation of hemoproteins such as hemoglobin, myoglobin, cytochrome P450 enzymes and mitochondrial respiratory cytochromes [2,4,8].

Heme biosynthesis is tightly regulated through different mechanisms. In the liver, regulation occurs through feedback control of the rate-limiting enzyme ALAS1. *ALAS1* gene expression is induced by various factors that increase heme demand, including stress and nutritional status, as well as medications and substances that activate the cytochrome P 450 system in the liver. Heme, on the other hand, exerts a negative feedback on ALAS1 activity via the suppression of *ALAS1* transcription, destabilization of *ALAS1* mRNA, repressed translocation of the enzyme into the mitochondrion, and direct inhibition of the ALAS1 enzyme activity [9]. Heme was also shown to induce the protease Lon peptidase 1, which plays a role in breaking down the ALAS1 enzyme [10]. In erythroid cells, heme biosynthesis is modulated primarily via the synthesis of the erythroid-specific enzyme ALAS2. *ALAS2* expression is regulated by erythroid-specific transcription factors, such as GATA1, as well as by the availability of iron via an iron-responsive element/iron regulatory protein binding system. While iron deficiency leads to inhibition of the *ALAS2* mRNA translation, increases in the intracellular iron levels cause degradation of the iron-responsive elements, which allows for the activation of *ALAS2* mRNA translation and in turn increases synthesis of the ALAS2 enzyme [11].

All porphyrias are caused by decreased activity of one of the enzymes in the heme biosynthetic pathway, with the exception of XLP, which is a consequence of gain-of-function variants in the *ALAS2* gene, leading to increased ALAS2 activity [12]. Nevertheless, the clinical presentation of the different types of porphyrias is distinct due to differences in pathophysiology, accumulating heme precursors or porphyrins, and affected tissues. Hence, the treatment and management of clinical manifestations also differ significantly, depending on the underlying condition. Historically, the avoidance of symptom-triggering factors and supportive management were the standard of care. Significant disease burden, impact on quality of life, comorbidities, chronic disease complications, and shortened life span are observed in all porphyrias. With growing knowledge of the disease mechanisms and advances in molecular medicine, progress has been made with respect to medical management, and recent developments as well as ongoing investigative approaches are reviewed in this manuscript (Table 2).

## 2. Acute Hepatic Porphyrias

The acute hepatic porphyrias (AHP) include AIP, VP, and HCP, which are caused by loss-of-function variants in specific enzymes of the heme biosynthetic pathway and have autosomal dominant inheritance. AIP is the most common of the AHPs with an estimated prevalence of approximately 5.9 patients with symptomatic AIP per 1,000,000 individuals. VP is thought to be half as common, and HCP is the rarest form of the autosomal dominant AHPs [25,26]. The fourth type of AHP is ADP, which has autosomal recessive inheritance, is characterized by a very severe clinical presentation and is extremely rare, with less than 10 cases reported worldwide [27].

It is notable that the autosomal dominant forms of AHP have decreased penetrance, and it is thought that less than 1% of all pathogenic variant carriers will remain asymptomatic throughout their life. The carrier frequency for pathogenic *HMBS* variants has been reported to be as high as approximately one in 1700 individuals, and the cause for the significant phenotypic variability even among carriers of the same pathogenic *HMBS* variant is unclear [28,29]. One report showed that penetrance may be higher in families with symptomatic AIP [30]. AIP is caused by deficiency of porphobilinogen deaminase (PBGD) or hydroxymethylbilane synthase (HMBS), the third enzyme in the heme biosynthetic pathway. The etiology for VP and HCP is decreased enzyme activity of protoporphyrinogen oxidase (PPOX) and coproporphyrinogen oxidase (CPOX), respectively [1]. The deficiency of these enzymes leads to accumulation of the heme precursors ALA and PBG, which are neurotoxic and responsible for the neurovisceral phenotype observed in the AHPs [31]. Precipitating factors that can trigger acute porphyria attacks including stress, excess alcohol intake, smoking, fasting, acute illness, steroids, hormonal preparations and other certain medications [32].

### 2.1. Clinical Presentation

Symptomatic AHP is characterized by acute neurovisceral attacks, which are characterized by diffuse abdominal pain accompanied by nausea, vomiting and constipation, back and chest pain, proximal muscle weakness and polyneuropathy. Tachycardia, hypertension and hyponatremia are often observed, and patients frequently report insomnia and anxiety. Cutaneous manifestations are not present in AIP or ADP but develop in up to 50% of individuals with VP and less frequently in HCP [2,33]. Acute porphyria attacks can either occur as an isolated episode or become recurrent in some individuals [34]. Long-term complications of AHP include hypertension, chronic pain, autonomic dysfunction, renal insufficiency, and in rare cases hepatocellular carcinoma [33,35,36].

The diagnosis of AHP is made by demonstrating significant elevation of urinary ALA and PBG levels. Determination of total porphyrins with fractionation in urine, feces, and plasma can aid in distinguishing between the different types of AHP (Table 2). With easier access to molecular analysis, genetic testing is often performed to definitively determine the specific type of AHP and identify other at-risk family members [32,37].

### 2.2. Contemporary Approaches to Management

The treatment of acute porphyria attacks consists of symptomatic therapy, including medication for pain control and nausea, seizure control, correction of electrolyte disturbances, hemodynamic stabilization and mechanical ventilation if indicated [32].

The only curative treatment approach for AHP is orthotopic liver transplantation (OLT), which has been shown to lead to rapid normalization of ALA and PBG levels and effectively prevent further acute porphyria attacks [38,39]. However, due to high morbidity and mortality, OLT is usually only considered as a last resort in individuals with very frequent recurring acute attacks who cannot be sufficiently well controlled using other treatment approaches. In an attempt to downregulate hepatic ALAS1 activity and subsequently decrease the production of ALA, PBG, and porphyrin metabolites, carbohydrate loading, dextrose infusion, and intravenous hematin administration are frequently used in individuals experiencing acute porphyria attacks [32,33,40]. Carbohydrates and glucose are thought to decrease ALAS1 activity by suppressing PGC1a and are usually less efficient than hematin, which directly inhibits ALAS1 activity by replenishing hepatic heme stores. Hematin is available as lyophilized hematin (Panhematin™, Recordati Rare Diseases, Northfield, IL, USA) in the US and heme arginate (Normosang™, Orphan Europe, Paris, France) in Europe. During acute porphyria attacks, three to four intravenous hematin treatments on consecutive days are often necessary to effectively lower elevated ALA and PBG concentrations and achieve symptom control [41,42,43]. Individuals who experience frequently recurrent acute attacks may benefit from off-label, regular prophylactic hematin infusions on a weekly or monthly basis in an attempt to regularly suppress increasing porphyrin precursor concentrations [33,44]. Complications of frequent hematin administration include damage to vasculature (the substrate can irritate the vessels and needs to be given through a large bore intravenous catheter or a central line) as well as chronic iron overload [45].

In 2019, the Food and Drug Administration (FDA) and, in 2020, the European Medicines Agency (EMA) approved givosiran (Givlaari™, Alnylam Pharmaceuticals, Cambridge, MA, USA), a synthetic small interfering RNA (siRNA) molecule that targets and downregulates *ALAS1* mRNA. The double-stranded siRNA molecule is conjugated to *N*-acetylgalactosamine (GalNAc) and administered subcutaneously in the form of a lipid nanoparticle, which allows for liver-specific delivery. After uptake into the hepatocytes, the siRNA molecules are trimmed into ~20 base pair strands by the endoribonuclease Dicer and subsequently further separated into single strands within the siRNA-induced silencing complex (RISC). Due to perfect base pair complementarity, the *ALAS1* mRNA is then targeted and cleaved by the argonaute-2 endonuclease, leading to effective knock-down of the gene [46,47].

In the phase I/II study, a significant decrease in *ALAS1* mRNA levels along with sustained near normalization of urinary ALA and PBG concentration was demonstrated with monthly givosiran injection [13]. This was confirmed in the phase III clinical trial, in which a significant reduction in the annualized acute porphyria attack rate (74% reduction in the treatment versus the placebo group) decreased the need for hemin use, and improved daily pain scores were observed with the monthly administration of givosiran at a dose of 2.5 mg/kg. Common side effects in the treatment group included injection-site reactions, nausea, rash, and fatigue. Notably, 15% of individuals treated with givosiran experienced transient elevation of liver transaminases, which led to treatment discontinuation in one trial participant. Furthermore, an increase in the serum creatinine level or reduction in eGFR was observed in seven (15%) individuals in the treatment cohort versus two (4%) in the placebo group. There was no correlation with baseline renal function, and resolution of the temporarily worsened kidney function over time without treatment discontinuation or dose adjustment was observed [14]. Real-world data collected after regulatory agency approval of givosiran shows an increased prevalence of hyperhomocysteinemia in individuals with AHP treated with the new siRNA drug. While increased homocysteine concentrations have been reported previously in AHP, initiation of therapy with givosiran seems to lead to a further increase in plasma levels, raising concern for potential cardiovascular or neurologic complications. It is hypothesized that heme depletion could interfere with cystathionine-beta-synthase, which is the enzyme primarily responsible for the conversion of homocysteine, although alternative mechanisms could also be involved. Supplementation with pyridoxal phosphate (vitamin B6) led to improved or normalized homocysteine levels in some cases [48,49,50,51].

### 2.3. Emerging Therapies

With recent advances in vector-mediated gene therapy for inherited conditions, this approach also seems promising for AHPs. A phase I clinical trial (NCT02082860) was conducted in Europe, using a recombinant adeno-associated vector expressing porphobilinogen deaminase (rAAV2/5-*PBGD*), the enzyme deficient in AIP [15]. In an open-label dose escalation study, a total of eight participants received a one-time intravenous administration of this vector construct in different doses. While a trend towards symptom improvement (with respect to hospitalizations and hemin use) was noted, no change in the concentration of the porphyrin precursors ALA and PBG was observed. While all participants developed neutralizing anti-AAV5 antibodies, no cellular immune response against the transgene or the virus capsid occurred. It is thought that even the highest dose of rAAV2/5-*PBGD* administered in this study was not high enough to achieve a therapeutic effect. This result prompted further investigational efforts to optimize the gene therapy approach by altering the *PBGD* sequence to create a protein with increased enzymatic activity without having to administer a higher vector dose. In addition, different approaches to decrease the immune system response and thereby improve the efficacy of rAAV-vector mediated gene therapy are underway [16,17].

In a different approach, human *PBGD* mRNA is packaged into liquid nanoparticles, which are administered intravenously and taken up by hepatocytes via receptor-mediated endocytosis. Preclinical studies showed dose-dependent protein production in an AIP mouse model, and rapid normalization of the porphyrin precursors was achieved during induced acute porphyria attacks. Repeat administration proved to be safe and efficacious, and the fact that safety has also been shown in non-human primates raises hope that this approach may also be translatable into humans [18].

## 3. Erythropoietic Cutaneous Porphyrias

Erythropoietic porphyrias present primarily with cutaneous photosensitivity and a distinct clinical presentation. These include the non-blistering porphyrias, EPP and XLP as well as CEP, which presents with blistering skin lesions.

### 3.1. Erythropoietic Protoporphyria and X-Linked Protoporphyria

EPP is the third most common porphyria overall and the most frequent type in children [1]. Prevalence estimates of EPP range from 1:75,000 in the Netherlands to 1:200,000 in the UK [25,52]. A recent study that analyzed exome sequencing data from the UK Biobank determined that the prevalence of EPP is 1.7–3.0 times higher than previously thought in the UK [53].

EPP is an autosomal recessive condition caused by deficiency of ferrochelatase (FECH), the last enzyme in the heme biosynthetic pathway, which catalyzes the insertion of iron into protoporphyrin to form heme. This enzyme also affects the insertion of zinc into those protoporphyrin molecules that are not used for heme formation, leading to the production of zinc protoporphyrin [4,54,55]. The FECH enzyme is encoded by the *FECH* gene, and to date, more than 190 pathogenic *FECH* variants causing significant protein instability and loss of enzymatic function have been described [56]. A common low-expression polymorphism (*FECH* IVS3-48C > T) affects splicing and leads to a decrease in enzyme activity of 20–30% [57]. The carrier frequency for this hypomorphic *FECH* allele varies among different ethnicities; it is present in up to 40% of Asians, approximately 10% of Caucasians, and very rarely present in individuals of African descent [58]. In most cases, EPP is caused by coinheritance of one copy of the low-expression allele and a pathogenic *FECH* variant *in trans*, which decreases the overall FECH activity to <35% of normal and results in photosensitivity. The presence of two pathogenic *FECH* variants is observed in approximately 5% of EPP patients [52,59]. Decreased FECH activity leads to the accumulation of protoporphyrin in bone marrow reticulocytes from where it enters the plasma via mature erythrocytes and is transported to the skin and liver [60]. Since FECH catalyzes the insertion of iron and zinc into protoporphyrin, the majority (>85%) of accumulating protoporphyrin is metal free [61,62].

XLP is caused by gain-of-function alleles in exon 11 of the *ALAS2* gene and is X-linked in inheritance. These *ALAS2* variants lead to activation of the erythrocyte-specific ALAS enzyme and result in the overproduction of protoporphyrin in excess of what is required for heme synthesis in bone marrow [63]. Since there is normal function of the FECH enzyme, a larger proportion of the accumulating protoporphyrin is zinc bound, and the rest is metal free (~50–85%) [12]. Given the X-linked inheritance, males are usually more severely affected, whereas the phenotype in females can be variable and range from asymptomatic to severe.

#### 3.1.1. Clinical Presentation

Cutaneous photosensitivity in EPP/XLP is caused by protoporphyrin presence in the small blood vessels of the skin, leading to photoactivation upon sun exposure. This phototoxic reaction results in swelling, itching, burning, erythema and severe pain in sun-exposed areas. Symptom onset usually first occurs in early childhood, and the condition persists throughout life. Bullous skin lesions, skin fragility and hirsutism are absent, and symptoms resolve after several days of sun avoidance without scarring. Most frequently affected areas include the dorsal aspect of the hands and the face [1,64]. Mild iron deficiency anemia may occur in some individuals with EPP/XLP, but hemolysis is typically absent [65]. Vitamin D deficiency is a common finding in EPP/XLP [66]. Hepatic involvement with elevated serum aminotransferases can be observed in approximately 20–30% of individuals with EPP/XLP, and in rare cases (~1–5%), rapidly progressive liver failure may occur [67,68,69]. Approximately 25% of EPP/XLP patients have been reported to form gallstones consisting of crystallized protoporphyrins [70].

#### 3.1.2. Contemporary Approaches to Management

Acute phototoxic reactions improve after several days of avoidance of sun exposure and cooling measures. Pain medication including narcotics is usually not efficient in alleviating pain. Antihistamines and steroids may improve symptoms, although a beneficial effect has not been clearly documented [71].

Sun avoidance and sun-protective clothing are the mainstay of EPP/XLP management. Tinted car windows and sunscreens containing zinc oxide or titanium dioxide help decrease sun exposure, and affected individuals often adjust their lifestyle to minimize sun exposure as much as possible [71]. Prophylactic treatment with oral beta-carotene can lead to mildly improved tolerance of sunlight if plasma levels are sufficiently high. This usually requires intake of high doses of beta-carotene, which tend to cause orange skin discoloration as an unpleasant side effect [72,73]. Other approaches, such cysteine, *N*-acetyl cysteine and vitamin C, have been tried but there are no data that show a beneficial effect on sun tolerance [74].

Afamelanotide (Scenesse™, Clinuvel Pharmaceuticals, Melbourne, Australia), an analogue of the human α-melanocyte stimulating hormone (α-MSH), was approved by the EMA in 2014 and by the FDA in 2019. Afamelanotide is administered in the form of a subcutaneous implant and binds to the dermal melanocortin-1 receptor, leading to increased production of the photoprotective substance eumelanin in the skin. In addition to producing a tan, eumelanin induces antioxidant activities, enhances DNA repair processes, and modulates inflammation [75,76]. Two multicenter, double-blind, placebo-controlled phase 3 clinical trials in Europe and the US with a total of 168 EPP/XLP patients showed increased pain-free time after sun exposure as well as a lower number of phototoxic reactions in the treatment versus the placebo group. In addition, patient-reported quality of life improved in participants who received afamelanotide. The most common side effect that could unequivocally be attributed to the afamelanotide implant was skin discoloration at the injection site. Other reactions such as nausea and nasopharyngitis were equally frequent in the placebo group [19,77].

In cholestatic liver disease, the use of cholestyramine and ursodeoxycholic acid has been described in an effort to increase protoporphyrin excretion [78,79]. In addition, plasmapheresis, red cell exchange transfusions and intravenous hemin administration have been trialed, but there is insufficient evidence to strongly support these measures [80,81]. For end-stage liver failure, orthotopic liver transplantation (OLT) is indicated. However, given the high risk of recurrent liver disease in EPP and the fact that protoporphyrin is produced in bone marrow, hematopoietic stem cell transplant (HSCT) has been reported as a curative approach either sequentially after OLT or as a primary intervention in cases without progressed liver fibrosis [79,82,83].

#### 3.1.3. Emerging Therapies

MT-7117 (Dersimelagon™, Mitsubishi Tanabe Pharma America, Jersey City, NJ, USA) is an orally administered small molecule that acts as a selective melanocortin-1 receptor agonist and increases dermal melanin production in the absence of ultraviolet radiation exposure, which is usually the main stimulus for melanin synthesis. In the recently completed multicenter, randomized, placebo-controlled phase 2 clinical trial (NCT03520036), which included 102 EPP/XLP individuals, placebo was compared to treatment with low-dose (100 mg daily) and high-dose (300 mg daily) MT-7117 [20]. The primary outcome measure was the change from baseline in relation to the average daily duration of sunlight exposure tolerated without symptoms, which were defined as the prodromal symptoms that frequently precede a phototoxic reaction and include tingling, itching, burning and stinging. Patients in both treatment groups showed a significant increase in average daily time (>50 min) to first prodrome at week 16 (100 mg group: *p* = 0.008; 300 mg group: *p* = 0.003). The overall side effect profile in the treatment groups was reported as favorable, with the most commonly reported events being nausea (27.9%), ephelides (23.5%) and skin hyperpigmentation (20.6%) [20].

A multicenter, randomized, double-blind, placebo-controlled phase 3 clinical trial (NCT04402489) assessing the same primary endpoint and measuring patient-reported outcomes regarding pain and physical function is currently underway.

The majority (~90%) of individuals with EPP carry the hypomorphic *FECH* polymorphism IVS3-48C > T, which leads to the increased use of a cryptic splice site between exons 3 and 4. This results in the transcription of unstable mRNA with a premature stop codon and, therefore, overall decreased ferrochelatase enzyme activity [57]. Gouya et al. identified a sequence within intron 3 that, when targeted by an antisense oligonucleotide (AON-V1), led to redirection from the cryptic to the physiologic splice site and increased the production of wild-type mRNA [21]. Transfection of lymphoblastoid cell lines derived from symptomatic EPP patients with AON-V1 resulted in increased production of functional *FECH* mRNA. In developing erythroblasts from an EPP patient, erythrocyte protoporphyrin IX (PPIX) concentration after adding ASO-V1 decreased to the level observed in an asymptomatic pathogenic *FECH* variant carrier but did not normalize [21]. Thus far, no data are available on using this approach in vivo, but the development of a nanocomplex in which AON-V1 is coupled to a bifunctional peptide that facilitates delivery of the AON construct to erythroid cells and helps prolong redirection of splicing towards the physiological splice site offers a promising outlook [22].

The exact etiology of microcytic anemia and iron deficiency is not known, and individuals with EPP/XLP seem to have normal iron absorption and hepcidin response [65,84]. Similarly, the role of iron supplementation in the treatment of EPP and XLP remains unclear. Based on single case reports, iron administration in patients with XLP seems to improve anemia as well as protoporphyrin levels and have a positive effect on liver disease [85]. In EPP, however, there are conflicting reports with some individuals experiencing improvement and others exacerbation of their photosensitivity upon iron supplementation [86,87]. A clinical trial (NCT 02979249) assessing a change in protoporphyrin levels after starting iron supplementation was recently conducted, but no data have yet been reported.

### 3.2. Congenital Erythropoietic Porphryia

CEP is extremely rare with approximately 250 described cases in the literature, affecting individuals of all ethnic backgrounds. It is caused by a deficiency of uroporphyrinogen III synthase (UROS), the fourth enzyme in the heme biosynthetic pathway, which is encoded by the *UROS* gene [88]. Inheritance is autosomal recessive, and there is broad phenotypic variability ranging from intrauterine demise due to non-immune hydrops fetalis to later-onset disease with mild cutaneous involvement. Deficiency of UROS leads to accumulation of the enzyme’s substrate, hydoxymethylbilane, which is then converted non-enzymatically into uroporphyrinogen I and subsequently coproporphyrinogen I. These porphyrin metabolites cannot be further metabolized since the next enzyme in the pathway, coproporphyrinogen oxidase (CPOX), is stereospecific for the isomer III coproporphyrinogen. Accumulation of the non-physiologic and phototoxic isomer I porphyrinogens ensues in erythroid precursors in bone marrow, where they undergo auto-oxidation to the corresponding porphyrins and cause damage to the erythrocytes, resulting in hemolysis [89,90]. Deposition of the photocatalytic and cytotoxic porphyrins in the skin leads to the cutaneous symptoms observed in CEP upon exposure to sunlight and other sources of long-wave ultraviolet radiation. The porphyrinogen I isomers are excreted in large amounts in urine and feces, resulting in pink to dark-reddish discoloration of the urine, which is often observed as early as in the newborn period [91,92,93].

In three male individuals with CEP, beta-thalassemia and thrombocytopenia, a pathogenic variant in the X-linked *GATA1* gene, was identified as an underlying cause. The pathogenic variant associated with the CEP phenotype is located in an area that is critical for the formation of an *N*-terminal zinc finger, which is thought to interfere with the binding affinity of the gene to the erythroid-specific *UROS* promoter region and thereby alter *UROS* gene expression [94,95].

#### 3.2.1. Clinical Presentation

The first sign in many individuals with CEP is reddish discoloration of urine, which frequently occurs in infancy or early childhood. Photosensitivity tends to be severe with vesicle formation in sun- or light-exposed areas. Blisters are prone to rupture and have a high risk of superinfection. The healing process frequently leaves scars, and deformities with loss of digits and facial features, such as eyelids, lips and ear and nose cartilage can occur (photomutilation). Thickening of the skin, hypertrichosis of the face and extremities and brown discoloration of the teeth (erythrodontia) are common [91,96,97]. If not protected from light exposure, eye involvement, such as necrotizing scleritis, keratoconjunctivitis, blepharitis and ectropion, can develop [98,99,100]. Most individuals with CEP experience chronic hemolytic anemia, which can be severe and require blood transfusion, splenomegaly, secondary thrombocytopenia and leukopenia. Porphyrin deposition in the bones can lead to demineralization and severe osteoporosis [101,102].

#### 3.2.2. Contemporary Approaches to Management

Protection from exposure to sunlight, ultraviolet light and light emitted by fluorescent sources is the most important aspect of the prevention of cutaneous manifestations. Skin blisters and lesions need to be addressed promptly to prevent secondary infections, which frequently require topical or systemic antibiotic therapy. Other complications, such as ophthalmologic manifestations, are treated symptomatically. Vitamin D supplementation is crucial to avoid osteopenia and osteoporosis [5,103].

If hemolytic anemia is severe, frequent blood transfusions may be necessary to maintain the hematocrit above 32%. Chronic hypertransfusion leads to suppression of erythropoiesis and simultaneously decreased porphyrin production, which can result in improved photosensitivity. However, complications of long-term blood transfusions, such as iron overload, can occur [104]. Splenectomy can be considered in individuals with significant splenomegaly, hemolytic anemia and pancytopenia [103]. The only curative approach in CEP consists of hematopoietic stem cell transplant (HSCT), which is usually reserved for very severe cases given the high risk of morbidity and mortality [105,106].

#### 3.2.3. Emerging Therapies

Similarly to EPP/XLP, the role of iron and iron metabolism in CEP is not yet completely understood. Recently, some CEP patients were reported to show an improvement in hemolysis and photosensitivity after successive phlebotomies or off-label treatment with an iron chelator, effectively inducing iron deficiency [107,108,109]. A beneficial effect of iron chelation on photosensitivity and hemolysis was recently demonstrated in a murine CEP model as well as in a human erythroid cell line from a CEP patient, in which inhibition of iron-dependent erythroid-specific ALAS2 expression and iron-responsive element-binding protein 2 led to decreased porphyrin production [110]. Thus far, the experience in humans is based on single observations and isolated case reports, and no clinical studies have been performed.

Millet et al. discovered that the known antifungal medication ciclopirox binds to the UROS protein and stabilizes its folded form, thereby effectively acting as a chaperone. In a CEP mouse model treated with ciclopirox, UROS enzyme activity was restored, and a decrease in uro- and coproporphyrinogen I in red blood cells as well as improvement of splenomegaly were measured. It was demonstrated that ciclopirox targets an allosteric site, which is removed from the active center, and there is no interference with the enzyme’s catalytic center. Given this mechanism, it is thought that ciclopirox would be suitable for approximately 75% of missense variants and would not have any effect on intronic variants or splicing defects [23]. While no clinical trials in individuals with CEP have been performed to date, a phase I study in patients with hematologic malignancy revealed that ciclopirox was well tolerated at low and medium doses and had a stabilizing effect on the malignant disease state. The mechanism behind this phenomenon is thought to be intracellular iron chelation and disruption of iron-dependent pathways, such as Wnt signaling, which results in decreased expression of the antiapoptotic gene *SURVIVIN* [111]. Even though iron chelation is thought to improve photosensitivity and hemolysis in individuals with CEP, this does not seem to be the mechanism through which the effect of ciclopirox is conferred in this condition since the mRNA expression of genes involved in the heme biosynthetic pathway remained unchanged in the CEP cell line treated with ciclopirox. In addition, the uroporphyrinogen I concentration in cells treated with ciclopirox was not influenced by large amounts of iron, which is possibly explained by the fact that ciclopirox has only weak affinity for iron binding and rather acts as a stabilizer of heme than a competitor for metal chelation [23]. Additional studies to improve the toxicity and pharmacologic profile of ciclopirox are underway in preparation for potential clinical trials in individuals with CEP [24].

## 4. Hepatic Cutaneous Porphyrias

Hepatic cutaneous porphyrias include Porphyria Cutanea Tarda (PCT) and Hepatoerythropietic Porphyria (HEP). PCT is the most common type of porphyria with a prevalence of approximately 50 per one million and an incidence of 2–5 per million [26]. Most cases of PCT are acquired (PCT I), and only around 20% of individuals with PCT carry a pathogenic variant in the *UROD* gene (PCT II), which acts as a predisposing factor [1,2]. HEP is very rare, with less than 100 cases described in the literature and is caused by bi-allelic *UROD* variants, leading to significantly decreased urodecarboxylase (UROD) enzyme activity [112]. The sporadic form (PCT I) develops in the setting of liver-specific inhibition of the UROD enzyme activity, which can occur in the presence of several susceptibility factors, such as *HFE* variants, excess alcohol consumption, hepatitis C, end-stage renal disease, HIV and hormonal influences. Decreased UROD activity leads to the accumulation of uroporphyrinogen as well as the intermediate metabolites hepta-, hexa- and pentacarboxylporphyrins, which are then oxidized and excreted as their corresponding porphyrins. Circulation of these porphyrins in the skin capillaries causes cutaneous symptoms upon sun exposure due to photoactivation. The penetrance of PCT II is very low, and typically, no familial clustering is observed [1,2,3].

### 4.1. Clinical Presentation

Individuals with PCT experience bullous lesions and fluid-filled vesicles, which easily rupture and heal with crusting and scarring. These findings are present in sun-exposed areas such as the face, neck, ears and dorsal aspect of hands and forearms. In addition, marked skin fragility, milia, hypertrichosis, hyperpigmentation and severe thickening of the skin occur frequently. A mild to moderate increase in serum aminotransferases is present in >50% of PCT patients, and ferritin levels are either normal or elevated. The clinical picture in HEP is characterized by symptom onset in early childhood and severe phototoxicity with blistering, scarring and in some cases photomutilations [113].

### 4.2. Current Management Approaches

Iron is known to play a significant role in the pathogenesis of PCT, and reduction in iron stores and hepatic iron content is the mainstay of symptomatic treatment. This can be achieved by serial phlebotomies or oral iron chelator therapy if phlebotomy is contraindicated or poorly tolerated. Antimalarial agents, such as hydroxychloroquine or chloroquine, at low doses are an effective alternative if phlebotomy is not an option, although the mechanism of action is poorly understood. Susceptibility factors should be addressed or removed as much as possible. The treatment of HEP is based mostly on photoprotection since neither phlebotomies nor low-dose antimalarial agents have been shown to improve symptoms [114,115].

### 4.3. Emerging Therapies and Investigations

Chronic hepatitis C is one of the most common susceptibility factors for sporadic PCT [116]. Since PCT symptoms are often more debilitating than manifestations of chronic hepatitis C, PCT treatment was recommended before starting therapy for hepatitis C. However, recent advances in the treatment of hepatitis C and availability of the highly effective direct-acting antiviral (DAA) medications may lead to a paradigm shift, since some case reports showed that PCT-directed therapy was unnecessary after the use of DAAs in patients with PCT and hepatitis C. A phase 2 clinical trial (NCT 03118674) is currently underway, evaluating if the treatment of individuals with chronic hepatitis C and PCT with ledipasvir/sofosbuvir (Harvoni™, Gilead Sciences, Foster City, CA, USA) leads to resolution of PCT determined by porphyrin concentrations, as well as clinical manifestations.

## 5. Conclusions and Future Directions

Porphyrias are a heterogeneous group of disorders characterized by phenotypic variability, chronic disability, and decreased quality of life. Treatment options have been limited with primarily symptomatic management. Curative approaches consist of hematopoietic stem cell transplant (in CEP and EPP) or liver transplantation (in AHP), which is a treatment of last resort for patients with severe, progressive disease who are at high risk for complications and mortality. There is a large unmet need for disease-modifying therapies for both acute and cutaneous porphyria. In recent decades, a better understanding of the underlying disease mechanisms as well as advances in molecular therapeutics have given rise to novel treatment approaches that are targeting the underlying pathomechanisms rather than merely addressing symptoms.

With further advances in the area of targeted therapies, more definitive or even curative treatment approaches, such as gene therapy, gene editing and alternate modes of gene or drug delivery, are being investigated for the different types of porphyria, with the goal to ameliorate the disease course as well as the quality of life of affected individuals.

## Figures and Tables

**Table 1:** Overview of the different porphyrias with respect to certain distinguishing key elements, affected enzymes, inheritance patterns and most common biochemical porphyrin findings.

Porphyria	Clinical Presentation	Tissue Site of Porphyrin Origin	Dysfunctional Enzyme	Inheritance	Most Significant Biochemical Findings *
**Acute Hepatic Porphyrias**					
ALA-dehydratase deficiency porphyria (ADP)	Acute neurovisceral	Hepatic	Delta-aminolevulinic acid dehydratase (ALAD)	AR	Urine: ALA, copro III Plasma: Zn-PPIX
Acute intermittent porphyria (AIP)	Acute neurovisceral	Hepatic	Hydroxymethylbilane synthase (HMBS)	AD	Urine: ALA, PBG, uro
Hereditary Coproporphyria (HCP)	Acute neurovisceral and cutaneous	Hepatic	Coproporphyrinogen oxidase (CPOX)	AD	Urine: ALA, PBG, copro III Feces: copro III
Variegate Porphyria (VP)	Acute neurovisceral and cutaneous	Hepatic	Protoporphyrinogen oxidase (PPOX)	AD	Urine: ALA, PBG, copro III Feces: copro III, PPIX
**Cutaneous Erythropoietic Porphyrias**					
Erythropoietic protoporphyria (EPP)	Cutaneous, rarely hepatic complications	Erythropoietic	Ferrochelatase (FECH)	AR	Plasma: free PPIX
X-linked protoporphyria (XLP)	Cutaneous, rarely hepatic complications	Erythropoietic	Delta-aminolevulinic acid synthase 2 (ALAS2)	X-linked	Plasma: free and Zn-PPIX
Congenital erythropoietic porphyria (CEP)	Cutaneous, hemolytic anemia	Erythropoietic	Uroporphyrinogen III synthase (UROS)	AR	Plasma & urine: uro I, copro I Feces: copro I
**Cutaneous Hepatic Porphyrias**					
Porphyria cutanea tarda (PCT)	Cutaneous	Hepatic	Uroporphyrinogen decarboxylase (UROD)	Sporadic, AD	Urine: uro, heptacarboxylporphyrin Feces: iso-copro
Hepato-erythropoietic porphyria (HEP)	Cutaneous	Erythropoietic, hepatic	Uroporphyrinogen decarboxylase (UROD)	AR	Urine: uro, heptacarboxylporphyrin Feces: iso-copro

*: Additional biochemical testing that can aid in establishing a diagnosis of porphyria includes plasma porphyrin measurement with fluorescence emission spectroscopy. If AIP or ADP are suspected, the determination of HMBS or ALAD enzymatic activity, respectively, can be performed to corroborate the diagnosis. Abbreviations: AR, autosomal recessive; AD, autosomal dominant; ALA, delta-aminolevulinic acid; copro, coproporphyrin; Zn-PPIX, zinc-bound protoporphyrin IX; PBG, porphobilinogen; uro, uroporphyrin.

**Table 2:** Overview of recently approved drugs, current clinical trials and emerging therapies for the treatment of the different types of porphyria. N/A = not applicable.

Porphyria	Recently Approved Drugs	Ongoing Clinical TRIALS	Emerging Therapies
**Acute Hepatic Porphyrias**			
**AIP, VP, HCP**	Givosiran (Givlaari™, Alnylam Pharmaceuticals, Boston, MA, USA), subcutaneous injection: siRNA targeting *ALAS1*, resulting in downregulation of *ALAS1* and decreased production of heme precursors ALA and PBG in addition to improved annualized acute porphyria attack rate [13,14].	**N/A**	Gene therapy with recombinant adeno-associated vector expressing porphobilinogen deaminase (rAAV2/5-*PBGD*), phase I clinical trial: no change in the concentration of ALA and PBG [15]. Optimization of gene therapy approach is being investigated [16,17].
			Human *PBGD* mRNA packaged into liquid nanoparticles, administered intravenously: proved to be safe and efficacious in non-human primates [18].
**Erythropoietic Cutaneous Porphyrias**			
**EPP, XLP**	Afamelanotide (Scenesse™, Clinuvel Pharmaceuticals, Melbourne, Australia), intracutaneous implant: α-melanocyte stimulating hormone analogue, leading to increased production of eumelanin and photoprotection [19].	MT-7117 (Dersimelagon™, Mitsubishi Tanabe Pharma America, Jersey City, NJ, USA), oral medication: selective melanocortin-1 receptor agonist, increased cutaneous melanin production. Phase 3 clinical trial (NCT04402489) currently underway [20].	Antisense oligonucleotide (AON-V1), increased production of functional *FECH* mRNA and, therefore, function protein with decreased PPIX concentration in cell cultures [21,22].
**CEP**	**N/A**	**N/A**	Ciclopirox: stabilizes UROS enzyme, improving catalytic function and leading to decreased porphyrin concentration in cell cultures and murine CEP models [23,24].
**Hepatic Cutaneous Porphyrias**			
**PCT**	**N/A**	Ledipasvir/sofosbuvir (Harvoni™, Gilead Sciences, Foster City, CA, USA), direct-acting antiviral used for treatment of chronic hepatitis C. Currently investigating time to resolution of PCT symptoms and porphyrin levels. Phase 2 clinical trial (NCT 03118674) currently underway.	**N/A**

## Data Availability

Not applicable.
